# Imported Cutaneous Diphtheria, United Kingdom

**DOI:** 10.3201/eid1003.030524

**Published:** 2004-03

**Authors:** Anne-Claire de Benoist, Joanne Margaret White, Androulla Efstratiou, Carole Kelly, Ginder Mann, Bernadette Nazareth, Charles James Irish, Deepti Kumar, Natasha Sarah Crowcroft

**Affiliations:** * Communicable Disease Surveillance Centre, London, United Kingdom; † Health Protection Agency, London, United Kingdom; ‡ Cambridgeshire and Peterborough Public Health Protection Unit, Huntington, United Kingdom; § Avon Health Protection Unit, Bristol, United Kingdom; ¶ Ealing Primary Care Trust, London, United Kingdom

**Keywords:** diphtheria, cutaneous diphtheria, disease outbreaks, immunization, prevention and control

## Abstract

Cutaneous diphtheria is endemic in tropical countries but unusual in the United Kingdom. Four cases occurred in the United Kingdom within 2 months in 2002. Because cutaneous diphtheria causes outbreaks of both cutaneous and pharyngeal forms, early diagnosis is essential for implementing control measures; high diphtheria vaccination coverage must also be maintained.

We report four cases of cutaneous diphtheria that occurred in the United Kingdom during a 2-month period in 2002 and review past cases.

## The Study

### Patient 1

In September 2002, a 6-year-old girl appeared for treatment with an infected mosquito bite on the ankle and chest lesions. The lesions developed while she was traveling with her mother and four siblings in Bangladesh. Her 12-year-old sister also had infected mosquito bites on her feet. Swab specimens, taken from the chest lesions and feet, were microbiologically cultured. *Staphylococcus aureus, Streptococcus pyogenes,* and toxigenic *Corynebacterium diphtheriae* var *mitis* were isolated from the specimens. The 6-year-old had received flucloxacillin for 5 days before the antimicrobial agent was changed to erythromycin. She had received three primary doses of diphtheria vaccine at birth, 5 months, and 9 months, and a booster vaccination in 2001. The 12- year-old was initially treated as a contact with 7 days of erythromycin; this treatment was extended to 14 days when the laboratory confirmed *C. diphtheriae* infection. She had received three primary diphtheria vaccine doses in 1994, but no booster. The children’s mother had received one dose of tetanus-diphtheria (Td) toxoid in July 2002, and their father, a household contact, had no immunization record.The remaining siblings had received at least three primary doses of diphtheria vaccine, and one had received a booster. Nose and throat swabs from all six members of the family, including the two patients, were negative for *C. diphtheriae*. The four contacts were given antimicrobial prophylaxis and completed immunization as appropriate. The two girls were kept home from school until their antibiotic regimen was completed and clearance swabs of the lesions taken 24 hours apart were confirmed as negative.

Ribotyping, a universal molecular typing method for bacteria based upon rRNA gene restriction pattern determination, was performed on the isolates from the two siblings. The ribotype patterns produced were indistinguishable from each other. The girls’ schools were asked for lists of all children who had been in contact with the two patients to establish their diphtheria immunization status as shown on the Child Health System (a population-based register of all children living in each locality, which includes information on vaccination status and other health indices). In addition, an information letter was sent to all parents.

### Patient 2

In September 2002, an 8-year-old Somalian girl, who had been in the United Kingdom for 4 months, was hospitalized with lesions on her legs and scalp, a sore throat, but no fever. She had no history of diphtheria immunization. The swabs from the throat and lesions yielded toxigenic *C. diphtheriae* var *mitis.* The organism isolated from the throat was only identified because the microbiology department screened all throat swabs routinely for corynebacteria. *S. aureus* was also isolated from the skin lesions. She was treated with flucloxacillin and penicillin. The skin lesions had been swabbed before September but had not been examined for *C. diphtheriae*. The isolates from the throat swab and the lesion were genotyped, and the ribotype patterns that resulted were identical. Two adults and five unvaccinated siblings were identified as contacts. After screening, all were negative for *C. diphtheriae* and were offered vaccination.

### Patient 3

At the end of October 2002, an 81-year-old man returned to England from Pakistan with an infected mosquito bite. Toxigenic *C. diphtheriae* var *mitis* was isolated from the lesion. The patient received diphtheria antitoxin and was treated with erythromycin and clarithromycin for 14 days. He had no history of diphtheria immunization. He had traveled alone to Pakistan. Twelve of his close contacts in the United Kingdom required microbiologic screening, although *C. diphtheriae* was not isolated from any. The contacts received erythromycin prophylaxis and were offered vaccine, except for two children who had already been vaccinated. Information also was sent to the patient’s contacts in Pakistan.

## Conclusions

In the United Kingdom, 17 patients with cases of cutaneous diphtheria due to toxigenic *C. diphtheriae* were reported from 1995 to 2002. All cases were travel-related (Table). Of 15 patients with a vaccination history, 6 were fully immunized (four primary doses by 5 years of age), 2 had received three doses of vaccine, and 7 had not been vaccinated.

**Table:** Distribution by year of cutaneous diphtheria cases caused by toxigenic *Corynebacterium diphtheriae* strains reported in the United Kingdom, 1985–2002

Y	Month	Sex	Age	Biotype	Specimen	Immunization status	Associated country
1985	Sept	M	5	*gravis*	Scalp	4 primary doses	Bangladesh
1990	Oct	F	19	*mitis*	Skin	Unvaccinated	Pakistan
1992	May	F	25	*mitis*	Foot. Insect bites	Unknown	Ghana
1992	July	F	8m	*mitis*	Skin	Unvaccinated	Bangladesh
1993	June	F	43	*mitis*	Mosquito bites	Unvaccinated	Gambia
1993	NA^a^	M	20	*gravis*	Skin (RTA)^b^	Unvaccinated	Pakistan
1994	NA^a^	F	64	*mitis*	Foot (leprosy)	Unvaccinated	Bangladesh
1994	Sept	F	32	*mitis*	Foot, throat (membrane)	4 primary doses	India
1995	March	M	27	*mitis*	Skin	4 primary doses	Thailand
1996	Nov	M	27	*intermedius*	Insect bites	4 primary doses	Nepal and Thailand
1997	Dec/Jan	M	39	*mitis*	Foot	4 primary doses + booster	Indonesia
1998	July	M	19	*mitis*	Leg wound	4 primary doses + booster	Tanzania
2000	Nov	F	44	*mitis*	Toe (diabetes)	Unknown	Gambia
2002	Sept	F	6	*mitis*	Chest	3 primary doses	Bangladesh
2002	Sept	F	12	*mitis*	Feet	3 primary doses	Bangladesh
2002	Oct.	M	81	*mitis*	Skin	Unvaccinated	Pakistan
2002	Sept.	F	8	*mitis*	Leg, scalp, throat	Unvaccinated	Somalia

^a^Not available. ^b^Road traffic accident.

In 1985, one patient with a secondary laryngeal case and a total of 16 carriers, including 8 who were secondary contacts of carriers, were associated with one cutaneous case. Dissemination to children and adults in several classes, schools, and households occurred within just 20 days (*1*). A patient in 1998 generated two asymptomatic carriers. The two sisters in 2002 acquired their lesions at approximately the same time, and thus we cannot determine whether they were infected by the same source or whether one infected the other.

High vaccination coverage is critical. The greater spread of infection after the 1985 case might be related to lower vaccination coverage at that time. In 1985, primary immunization for diphtheria was 85% compared to the current rate of 94%.

Cutaneous diphtheria, still endemic in tropical countries, is the most common nonrespiratory clinical manifestation of infection due to toxigenic isolates of *C. diphtheriae* (*2*). The disease is characterized by shallow skin ulcers, which can occur anywhere on the body and are usually chronic. They are often associated with infected insect bites, frequently coinfected with pathogens such as *S. aureus* and *S. pyogenes*. Systemic toxic manifestations are uncommon among immunized persons. Skin lesions absorb toxin slowly and can induce high levels of antibodies that produce natural immunization. These lesions are an important reservoir of infection and can cause respiratory and cutaneous infections in contacts as well as outbreaks (*3*). In several outbreaks, secondary transmission has been higher in contacts of patients with cutaneous infection than in those with respiratory tract infection. Cutaneous diphtheria may also cause greater environmental contamination, through dust and fomites (*4*).

Cutaneous diphtheria is still being reported in the United Kingdom, even in vaccinated patients and despite high diphtheria vaccination coverage. All cases so far have been acquired in countries where diphtheria is endemic. With increasing travel to and from these countries, more cases may occur. The potential for secondary transmission leads to a large number of contacts requiring follow-up, especially children at school. Moreover, cutaneous diphtheria is likely to be diagnosed less quickly than respiratory infection because the clinical appearance is nonspecific, and other pathogens often coinfect the lesions. Thus, we need to increase the awareness of clinicians and microbiologists of the importance of obtaining swab specimens from any chronic nonhealing skin lesions in patients who have traveled to a disease-endemic area. Wound swab samples from these patients should be examined for *C. diphtheriae.* Early diagnoses and reporting are crucial to trigger effective public health control measures (*5*).
